# Towards an Individual Differences Perspective in Mindfulness Training Research: Theoretical and Empirical Considerations

**DOI:** 10.3389/fpsyg.2020.00818

**Published:** 2020-05-19

**Authors:** Rongxiang Tang, Todd S. Braver

**Affiliations:** Department of Psychological and Brain Sciences, Washington University in St. Louis, St. Louis, MO, United States

**Keywords:** mindfulness training, meditation, individual differences, personality traits, disposition, personalized medicine, precision medicine

## Abstract

A growing body of research indicates that mindfulness training can have beneficial effects on critical aspects of psychological well-being, cognitive function, and brain health. Although these benefits have been generalized to the population level, individual variability in observed effects of mindfulness training has not been systematically investigated. Research on other similar forms of psychological intervention demonstrates that individual differences are prominent in terms of intervention responsiveness and outcomes. Furthermore, individual characteristics such as personality traits have been shown to play a crucial role in influencing the effects of intervention. In light of these lines of evidence, we review representative work on individual differences in mindfulness training and advocate for an individual difference perspective in mindfulness training research. We discuss relevant empirical evidence of individual differences potentially influencing behavioral outcomes of mindfulness training, focusing on both cognitive function and psychological well-being. Finally, theoretical considerations and potentially fruitful research strategies and directions for studying individual differences in mindfulness training are discussed, including those involving cognitive neuroscience methods.

## Introduction

Mindfulness training (or mindfulness-based intervention) has garnered popularity both within the scientific community, and among the lay public, as an effective intervention that promotes health and well-being. Adapting practices and techniques from Eastern Buddhist traditions, mindfulness training was first introduced to Western culture in the 1970s as a set of secular and contemplative practices that alleviate stress and psychological distress (Kabat-Zinn, 1990). Subsequent development of these practices has given rise to many different yet similar mindfulness training programs, which all have the same goal of promoting attention to and awareness of present moment experiences with a non-judgmental and accepting attitude (Hölzel et al., 2011). Over the past few decades, a growing body of research has indicated that mindfulness training can have beneficial effects on critical aspects of psychological well-being, cognitive function, physiology, and brain health (Grossman et al., 2004; Chiesa et al., 2011; Gu et al., 2015; Tang et al., 2015). Currently, mindfulness training not only serves an active role in clinical treatment for patients with emotion-related disorders (Khoury et al., 2013), but has also become part of a healthy lifestyle/wellness approach adopted by individuals without a clinical diagnosis, who seek to enhance their psychological well-being and quality of life.

Nonetheless, this widespread application of mindfulness training deserves careful scrutiny. Most of the extant literature on mindfulness training has generalized its benefits to the population level, assuming homogeneous effects of training for every individual based on group-averaged results. However, this one-size-fits-all model is in tension with existing theoretical frameworks and empirical evidence that have been amassed with regard to individual variability in response to psychological interventions (Caspi and Bell, 2004; Gully and Chen, 2010). Furthermore, overlooking individual differences when evaluating the outcomes of intervention may inadvertently incur negative consequences, in that individual differences may potentially be contributing to several non-replications or mixed findings of previously observed mindfulness training benefits in cognition and psychological well-being (MacCoon et al., 2012, 2014; Rosenkranz et al., 2013). This is because group-averaged effects will be subject to fluctuation among samples with different distribution and variability (i.e., some samples may contain more individuals who would respond to the intervention than others). From a practical standpoint, considering individual variability in training responsiveness is critical for the application of mindfulness training both in clinical settings and in public domain, as this would enable a better understanding of the specific subgroups of individuals for whom the training would be most or least effective in achieving the desirable outcomes.

Indeed, the Precision Medicine Initiative announced by the U.S. government directly highlights the importance of tailoring prevention and intervention programs based on individual characteristics, in order to achieve better outcomes (Collins and Varmus, 2015). Consequently, it calls for more systematic investigation to identify and understand characteristics responsible for differential intervention effects across individuals. For mindfulness training, the motivation to adopt an individual difference perspective both in research and in application becomes increasingly apparent. As a relevant and related domain, psychotherapy research has made rapid progress in identifying potential moderators of intervention effects (Kraemer et al., 2002; Kraemer et al., 2006; Kazdin, 2007; Wallace et al., 2013). Although the effect sizes of these moderators are relatively modest, most studies have found individual characteristics to play a key role. For instance, personality traits have been shown to affect the degree to which depressive patients benefit from cognitive behavioral therapy (CBT) (Bagby et al., 2008). However, systematic investigations focusing on individual differences are relatively sparse in the field of mindfulness training. Perhaps since mindfulness training research is still in its infancy, much attention has been devoted to understanding its basic effects and mechanisms in changing brain and behavior, but less so on the potential individual variability present in these training-related effects.

Nonetheless, encouraging and emerging efforts have been made to advance our understanding of individual differences in domains such as personality traits (de Vibe et al., 2015; Nyklíček and Irrmischer, 2017), psychological well-being (Greeson et al., 2015; Gawrysiak et al., 2016), cognitive functions (Mrazek et al., 2013), and their respective roles in influencing mindfulness training effectiveness and outcomes. Rather than providing an exhaustive or comprehensive review of the literature, the goal of this paper is instead to provide case-study illustrations that highlight relevant domains of individual difference that can impact mindfulness training. Moreover, we propose useful research strategies and directions to point the field to this promising and fruitful research area, advocating for an individual differences perspective in scientific investigations of mindfulness training. We hope that this discussion will encourage and facilitate further rigorous and systematic investigations of individual differences in mindfulness training. The first section of this paper focuses on personality and dispositional traits, while the second and third sections describe how pre-existing individual differences in emotion regulation and cognitive abilities could influence training effects and outcomes. Finally, the fourth section discusses theoretical considerations and challenges, as well as relevant promising research strategies and future directions, including those involving cutting-edge cognitive neuroscience methods. We provide a table that summarizes key details of the studies discussed in these sections (see Table 1).

**TABLE 1:** Studies of individual differences in intervention and outcomes.

Study	Study design	Intervention	Sample size	Individual difference measures	Outcome measures
Bagby et al., 2008	RCT	CBT/PHT	146/129	Neuroticism – Revised NEO Personality Inventory	Depression severity –Hamilton Depression Rating Scale
de Vibe et al., 2015	RCT	MBSR/waitlist	144/144	Neuroticism, Conscientiousness – Basic Character Inventory	Psychological distress – The General Health Questionnaire Subjective well-being – Subjective Well-being Scale Student stress – Perceived Medical School Stress
Nyklíček and Irrmischer, 2017	Longitudinal	MBSR	167	Neuroticism – Revised NEO Personality Inventory	Anxiety – Profile of Mood States
Krick and Felfe, 2019	RCT	Mindfulness intervention/education course	126/141	Neuroticism, Openness – NEO Personality Inventory	Self-care – Health-oriented Leadership Instrument Psychological strain – Irritation Scale Negative affect – Positive and Negative Affect Schedule-X Heart Rate Variability
Ding et al., 2015	RCT	Mindfulness intervention/relaxation training	42/42	Neuroticism, Extraversion – Eysenck Personality Questionnaire Depression, Fatigue, Anger – Profile of Mood States	Creativity – Torrance Test of Creative Thinking
Talbot et al., 2003	Quasi-experimental	WSIR/TAU	48/38	Agreeableness, Extraversion – NEO Personality Inventory	Psychological distress symptoms – The Symptom Checklist-Revised
Barkan et al., 2016	Longitudinal	MBSR	100	Openness, Agreeableness – NEO Personality Inventory	MBSR Utilization – Weekly Logs
Shapiro et al., 2011	RCT	MBSR/waitlist	15/15	Trait Mindfulness –Mindful Attention Awareness Scale	Trait mindfulness – Mindful Attention Awareness Scale
Shahar et al., 2010	RCT	MBCT/waitlist	26/19	Trait Mindfulness –Mindful Attention Awareness Scale	Depression symptoms – Beck Depression Inventory
Studer-Luethi et al., 2012	RCT	WMT/control	47/43	Conscientiousness – Mini-Marker Set	Working Memory – N-back training level
Mrazek et al., 2013	RCT	Mindfulness intervention/nutrition class	26/22	Mind wandering – Task Unrelated Thoughts	Working Memory – Operational Span Task

*CBT, Cognitive Behavioral Therapy; PHT, Pharmacotherapy; MBSR, Mindfulness based stress reduction; WSIR, Women’s Safety in Recovery; TAU, Treatment as usual; WMT, Working memory training.*

## Could Personality and Dispositional Traits Influence Mindfulness Training Effects?

Personality and dispositional traits are the most frequently assessed dimension of individual difference, and are also popular moderators of intervention effects in psychotherapy research. Accumulating evidence demonstrates that personality traits can predict differential targeted behavioral changes in psychotherapy (Chapman et al., 2014). In the following section, traits that could play an important role in influencing mindfulness training effectiveness will each be identified and discussed based on supporting theories and empirical evidence from prior literature. Because the goal of individual differences investigation should always be confirmatory, rather than exploratory, it is critical to formulate *a priori* hypotheses regarding putative variables associated with differential intervention effects (Caspi and Bell, 2004). Employing such a theoretically oriented and hypothesis-driven approach would greatly mitigate the concerns of spurious statistical associations that can result from exploratory, “fishing expedition”-type analyses in which a myriad of putative dispositional traits are queried.

### Big Five Dimensions of Personality

The Big Five dimensions of personality have been theoretically hypothesized and empirically shown in previous work to influence the extent to which individuals benefit from psychological intervention including mindfulness training. According to several theoretical frameworks relating personality traits to intervention outcomes (Anderson, 1998; Chapman et al., 2014), all five dimensions of the Big Five theory have relevance to intervention effectiveness. Here, we extend this framework to discuss how the Big Five dimensions of personality could have an impact on mindfulness training.

#### Neuroticism

Neuroticism reflects a level of affective instability, in terms of anger, depression and other forms of negative affect, which is generally associated with low responsiveness to a wide range of psychological interventions. This is thought to be the case because high levels of neuroticism are frequently related to difficulties in intervention adherence (Anderson, 1998). One randomized controlled trial (RCT) study comparing the effectiveness of CBT and pharmacotherapy (PHT) did suggest that for patients (*N* = 275) with major depressive disorder, higher neuroticism was associated with more improvement in depressive severity post-treatment, if PHT was administered, but not if CBT was used (Bagby et al., 2008). However, such moderating effects of neuroticism seem to be dependent on the population group. In a recent RCT study of 288 students who underwent either 8 weeks of MBSR or served as waitlist controls, neuroticism was examined as one of the moderators of mindfulness training effects; individuals with higher level of neuroticism exhibited greater improvement in subjective well-being and psychological distress (de Vibe et al., 2015). Likewise, in a longitudinal study of 167 participants with general stress complaints, individuals with higher neuroticism level showed a greater reduction in anxiety symptoms after 8-weeks of MBSR. Although controlling for baseline anxiety levels reduced this immediate effect, it still revealed a larger reduction in anxiety levels among such individuals between post-intervention and the 3-month follow-up (Nyklíček and Irrmischer, 2017). Similarly, in a RCT study of 267 police officers who underwent either mindfulness-based intervention or education course, higher neuroticism was also found to be positively related to improvement in self-care, as well as reduction in negative affect and psychological strain after 6 weeks (Krick and Felfe, 2019). These studies not only indicate the high utility of neuroticism in predicting psychological outcomes of mindfulness training, but also suggest that at least in populations with sub-clinical levels of anxiety and depression, high baseline neuroticism may actually lead to greater beneficial outcomes associated with mindfulness training.

In addition to the effects of neuroticism on psychological outcomes, one study investigated the moderating effects of personality traits and mood states on enhanced creativity following mindfulness practice. In a RCT study, 84 undergraduate students participated in either a week of 30 min daily mindfulness practices or relaxation practices. It was found that lower depression, lower fatigue, and higher anger at baseline predicted enhanced creative performance after mindfulness training (Ding et al., 2015). Furthermore, there were interactive effects of mood states and personality traits observed, such that individuals with lower neuroticism or extraversion exhibited greater improvement when they also reported a more positive and aroused mood (Ding et al., 2015). These predictors together explained 57% of variance in creativity improvement, suggesting not only that the extent of improvement was largely influenced by individual differences in baseline personality traits and mood states, but also that these pre-existing differences in individual characteristics should be important components to consider in understanding the mechanism of mindfulness interventions. However, this study offered a different perspective on the role of neuroticism; that is, lower neuroticism was associated with greater improvement in cognitive outcome, which is somewhat contrary to what have been found with regard to other psychological outcomes. Consequently, it suggests a need to further elucidate the exact role and mechanisms of personality traits in affecting different outcomes of mindfulness training in different populations.

Although it is unclear as to why neuroticism could have distinct effects on mindfulness training outcomes, one likely explanation is that for psychological outcomes, mindfulness training may work by improving the suboptimal emotion regulation and self-awareness capacities in individuals with high neuroticism, which together help them better cope with stress and negative affect (Hölzel et al., 2011). We believe that neuroticism is likely to be a personality trait that could have influence over a majority of psychological interventions, and thus could also be a promising target trait for future mindfulness investigations. However, more studies are needed to determine and confirm: (1) if higher neuroticism generally leads to better outcomes in psychological well-being or symptoms for populations without clinically diagnosed anxiety and depression disorders; and (2) if neuroticism is useful in predicting other mindfulness training-related behavioral outcomes, such as enhanced cognitive function.

#### Agreeableness and Extraversion

Although these two personality dimensions may not seem to be intuitive moderators of mindfulness training outcomes, evidence from other psychological interventions suggest that these traits could still be relevant. A quasi-experimental study conducted among 86 women with childhood sexual abuse histories found that women low in both agreeableness and extraversion had better treatment outcomes in self-report symptoms of psychological distress if they received a highly structured and skill-focused intervention – Women’s Safety in Recovery (WSIR), which focuses on problem solving exercises – rather than treatment as usual (TAU) devoted largely to group therapy, in which general strategies of crisis resolution and symptom reduction are discussed (Talbot et al., 2003). According to the Big Five framework, extraversion tends to manifest as talkative and outgoing behavior that facilitates interpersonal interaction and relationship (McCrae and Costa, 1991). Prior work suggests that extraverted individuals may prefer an intervention approach involving interpersonal interactions, while introverted individuals may prefer a more structured and goal-directed approach (Sanderson and Clarkin, 1994; Anderson, 1998). Similarly, agreeableness entails levels of friendliness, warmth and cooperativeness (McCrae and Costa, 1991), and has also been shown to associate with preferences for interpersonal approaches and group therapy during intervention (Bliwise et al., 1995; Anderson, 1998). Taken together, these results suggest that agreeableness and extraversion could potentially be key players in influencing the outcomes of group-based interventions, perhaps through preferences in intervention approaches. A practical implication of these personality-related preferences is that psychological interventions might be rendered more effective if they were administered in the manner that individuals prefer.

For mindfulness-based intervention, such as the most popular variants of mindfulness training (e.g., MBSR), group discussion and activities are involved in almost every class session (Santorelli et al., 2017). It is possible that those low in agreeableness and extraversion may find highly structured, one-on-one, and/or skill-focused mindfulness training more appealing than programs that involve regular group discussion and engagement to acquire the mindfulness skills, as these individuals are likely to feel intimidated in such group scenarios. However, it should also be noted that agreeableness has the potential to influence mindfulness training outcomes through a completely different mechanism. One study of 100 older adults who underwent 8 weeks of MBSR did find that individuals with high agreeableness tended to practice more mindfulness during the period of intervention than those low in agreeableness, but did not continue to show more practice after intervention completion (Barkan et al., 2016). This finding is particularly interesting as it suggests that individuals with high agreeableness may be more cooperative and compliant to instructor’s instructions of completing daily practice outside of the class, but less so when no instructions were given after intervention completion (Barkan et al., 2016). Although the study did not directly investigate whether or not varying amount of practice time was associated with differential training effects on outcomes of interest, previous research in mindfulness training has suggested a positive relationship between practice time and improvement in various outcome measures, such as cognitive function and brain functional connectivity (Chan and Woollacott, 2007; Jha et al., 2010; Brewer et al., 2011). Overall, this preliminary evidence suggests that it is worth considering extraversion and agreeableness as moderators of intervention effects in future investigations of mindfulness training.

#### Openness to Experience

Openness to experience represents a sense of curiosity, acceptance and open-mindedness to diverse experiences. This is highly relevant for mindfulness training, since individuals high in openness to experience may be more likely to seek out and practice complementary and alternative medicine (CAM) techniques, such as yoga and mindfulness-based practices in daily life (van den Hurk et al., 2011; Thomson et al., 2014). As evident from one study (mentioned briefly above) exploring the influence of personality traits on the frequency of practicing mindfulness techniques, individuals with high openness to experience were found to engage in more mindfulness practices in their daily life, both during and after an 8-week mindfulness intervention program (Barkan et al., 2016). The results held even when demographic differences, such as age, educational level, and sex were statistically controlled, suggesting the unique impact of openness to experience on practice behavior. Relatedly, openness to experience was found to moderate heart rate variability response in police officers following 6-week of mindfulness-based intervention, such that individuals with high openness to experience exhibited more improvement in heart rate variability (Krick and Felfe, 2019).

Based on these findings, openness to experience seems to have a special relevance to mindfulness training, as this trait is likely to determine how much individuals would engage in the actual practices that may lead to observable improvement in any training-related outcomes. This is not surprising since previous evidence suggests that individuals high in openness tend to benefit more from approaches focusing on self-exploration and discovery (Anderson, 1998) and they are also more likely to be curious about their internal experiences (Barkan et al., 2016), leading to further exploration and practice of mindfulness techniques involving self-awareness and interoception. This hypothesis also poses both a challenge and an interesting opportunity for future mindfulness training research; that is, how to adequately account for the effects of openness in influencing training outcomes in volunteer participants who get compensated for their participation, but may nonetheless have an inherent preferred bias toward mindfulness training.

#### Conscientiousness

Conscientiousness has long been implicated in intervention adherence and compliance, since it encompasses qualities such as persistence, self-control, industriousness and a sense of responsibility that facilitate such behavior (Sanderson and Clarkin, 1994; Chapman et al., 2014). A nationwide study of conscientiousness has demonstrated that this personality dimension is positively related to treatment adherence, potentially leading to different health outcomes (Hill and Roberts, 2011). Relatedly, a meta-analysis found that conscientiousness is positively related to motivation to learn, which in and of itself correlates with intervention effectiveness (Colquitt et al., 2000). This seems to suggest that motivation may be one of the underlying factors contributing to adherence to intervention instructions and protocols in high conscientious individuals, which may ultimately manifest as improvement in intervention outcomes. In a study of cognitive training of working memory (*N* = 47), high conscientiousness was positively associated with overall improved working memory performance at post-training in conjunction with high training enjoyment reported by participants (Studer-Luethi et al., 2012). This finding further corroborates the notion that high conscientious individuals are more motivated to commit to the goals of training and may also find it more enjoyable to comply with training instructions (Komarraju and Karau, 2005).

However, conscientiousness has been associated with mixed results when examined as a moderator of mindfulness training outcomes. For example, in the abovementioned study where neuroticism and openness to experience were found to separately moderate psychological and physiological outcomes, conscientiousness showed no significant moderating effect (Krick and Felfe, 2019). Furthermore, conscientiousness also had no association with practice time in the study that found two other dimensions (agreeableness and openness) to be related to practice time (Barkan et al., 2016). Nonetheless, in the previously mentioned study of MBSR effects on psychological outcomes, individuals with high conscientiousness showed greater reduction in stress after intervention (de Vibe et al., 2015), suggesting that conscientiousness may be a moderator of intervention effects. Therefore, while conscientiousness is likely to indirectly impact the effectiveness and outcomes of most interventions, including mindfulness training, through its interaction with other mental processes such as motivation to learn and adherence, further research is needed to address these mixed findings and elucidate precisely what behavioral outcomes of mindfulness training are most likely to be moderated by conscientiousness.

### Trait Mindfulness

Finally, there is one additional noteworthy dispositional trait unique to mindfulness training effectiveness and outcomes. Trait mindfulness generally refers to the innate ability to focus attention on present moment experiences and to maintain present moment awareness with a non-judgmental and non-reactive attitude (Brown and Ryan, 2003). There are multiple questionnaires for assessing trait mindfulness; two of the most popular are the Mindful Attention Awareness Scale (MAAS) (Brown and Ryan, 2003) and the Five Facet Mindfulness Questionnaire (Baer et al., 2008). Some theorists hypothesize that mindfulness programs which repeatedly induce mindfulness states may lead to more stable and trait-level changes in mindfulness (Kiken et al., 2015). Not surprisingly, one RCT study conducted in 30 people (15 in each group) showed that individuals who underwent an 8-week MBSR significantly enhanced trait mindfulness compared to those in a control group, and that people with high trait mindfulness at baseline exhibited a greater increase in trait mindfulness at post-intervention (Shapiro et al., 2011). Despite the preliminary nature of this finding, individual differences in trait mindfulness seem to be highly relevant to predicting mindfulness training effects on psychological well-being, since trait mindfulness is positively related to self-report measures of psychological health (Brown and Ryan, 2003; Keng et al., 2011).

The positive correlations between individual differences in trait mindfulness and cognitive performance in attention and memory observed in previous studies (Schmertz et al., 2009; Anicha et al., 2012; Ruocco and Direkoglu, 2013), suggest that trait mindfulness could also exert an influence over the effects of mindfulness training on cognitive function. Specifically, there is some evidence illustrating that trait mindfulness may potentially mediate the effects of mindfulness training on cognitive function, by simultaneously reducing emotional interference and enhancing attentional control to foster optimal performance (Ortner et al., 2007; Shahar et al., 2010). For example, in an RCT study of 45 patients with recurrent depression, patients who received 8 weeks of mindfulness-based cognitive therapy (MBCT) showed an improvement in trait mindfulness, which also mediated the reduction in depressive symptoms at post-intervention, suggesting that trait mindfulness is not only an outcome of MBIs with pre-existing individual differences, but is also a critical mediator of affective outcomes associated with MBIs (Shahar et al., 2010). Likewise, one recent study (*N* = 60) showed that the positive relationship between trait mindfulness and executive attention performance is mediated by the brain P300 event-related potential component, as assessed via electroencephalography (EEG), suggesting a neuropsychological basis for such relationship (Lin et al., 2019). Thus, trait mindfulness could well be one of the most important contributors to differential training effects, as well as a vital player in the underlying mechanisms and processes supporting training-related improvement in cognition and mental health. However, there is still a poor understanding of the directionality of the effects of trait mindfulness on different training outcomes. Indeed, studies of personality traits seem to indicate the contrary directional pattern, in that individuals with lower psychological well-being (and also likely to have lower trait mindfulness) at baseline are those who tend to experience more improvement in psychological outcomes.

Overall, there is sufficient evidence to support the assumption that individual differences in the Big Five personality traits and trait mindfulness will contribute to the extent to which individuals benefit from mindfulness training. Additionally, it is evident that the influence of some of the dispositional traits may not be limited to a particular psychological intervention or outcome. Lastly, these individual differences variables may interact with global level processes and mechanisms associated with affecting habitual behavioral and psychological patterns, which in turn could contribute to differential effects on outcomes of interest in individuals who undergo some form of intervention (Anderson, 1998; Chapman et al., 2014).

## Could Pre-Existing Differences in Emotion Regulation Affect Mindfulness Training Outcomes?

Emotion regulation, a core psychological process thought to be critical for maintaining well-being, has also been associated with considerable individual variability in terms of preferred regulatory strategies and styles (Gross and John, 2003). Conceptually, emotion regulation refers to the process of exerting control over one’s emotion through a wide range of strategies to influence the type of emotions that one has, experiences, or expresses (Gross, 2001). Previous literature has suggested that successful and effective emotion regulation is achieved by engaging adaptive strategies that often take place early on in the trajectory of individual’s emotional experience (Peña-Sarrionandia et al., 2015). Moreover, emotion regulation is often targeted as a key component of mindfulness training (Tang et al., 2015). In this section, we focus on a well-established theoretical model of emotion regulation to discuss how individual differences in two specific emotion regulation strategies may impact mindfulness training outcomes.

### Cognitive Reappraisal and Expressive Suppression

According to Gross’s influential model of emotion regulation, individuals differ in terms of how they control emotion by employing two different regulatory strategies: cognitive reappraisal and expressive suppression (Gross and John, 2003). Cognitive reappraisal refers to a form of cognitive process that reconstructs an emotionally aroused situation in a way that reduces its emotional impact, which typically occurs before the emotional response has been fully generated (Gross and John, 2003). For instance, one might perceive a job interview failure as an opportunity to learn from experience, rather than as a test of one’s worth. In contrast, expressive suppression is a form of modulatory process that inhibits an ongoing emotional response or expression, which primarily modifies the behavioral expression of the emotion, but leaves the internal emotional experience intact (Gross, 2001). For example, a person might be offended by another individual and feel angry at the moment, but then decide to suppress their emotion, so as not to express it externally.

Each of these two strategies can result in significant affective consequences for individuals’ well-being. Theoretical models and empirical research suggest that reappraisal is generally more effective than suppression, and that individuals who engage in habitual suppression experience more negative emotion, whereas people who employ reappraisal experience more positive affect and have better psychological health (Gross and John, 2003). With regard to mindfulness training, these pre-existing differences in emotion regulation tendencies could lead to differential training effects on psychological well-being, and even cognitive function, by interacting directly with the training process. Typical mindfulness practices involve observing and attending to any emotion, thoughts, or sensations that arise at the present moment. Such attentive observation of emotional experiences is achieved with a non-emotionally reactive and non-judgmental attitude, which may partly require the engagement of cognitive reappraisal to translate emotionally aroused experiences into emotion-detached events (Hölzel et al., 2011). One recent longitudinal study (*N* = 339) of an 8-week of mindfulness intervention further confirms the notion that mindfulness training not only elicits cognitive reappraisal during training, but also increases self-reported positive reappraisal at post-training (Garland et al., 2011). Conversely, suppression is discouraged during mindfulness practices, as the goal of these practices is to openly accept any emotional experiences, regardless of their pleasantness or unpleasantness.

As such, cognitive reappraisers may likely find mindfulness training more intuitive than expressive suppressors, since the latter often regulate their emotion by forcefully suppressing it. This difference could entail two possibilities (not necessarily mutually exclusive) for mindfulness training effects on psychological well-being: (1) cognitive reappraisers would show more improvement since they can easily acquire and practice mindfulness techniques involving reappraisal for emotion regulation, thereby leading to better psychological outcomes and cognitive function; or (2) expressive suppressors would exhibit more enhancement in psychological health and cognition, especially in positive affect, since they could gradually shift away from the previous mentally taxing strategy, to a more beneficial and effective way of emotion regulation through recurrent mindfulness practices. Although there has not been empirical research specifically examining these two hypotheses, the abovementioned inter-relationships among emotion regulation, psychological well-being, and mindfulness training suggest that the tendency for either reappraisal or suppression should theoretically be relevant for influencing the extent of mindfulness training effects on psychological health and cognitive function.

Taken together, individual differences in styles of emotion regulation could potentially interact with intervention processes, by influencing the state of learning and acquisition of relevant techniques to induce differential effects at the individual level. It should be noted that the putative emotional regulatory strategies mentioned above are by no means exhaustive, but they do serve as useful examples to illustrate the importance of examining pre-existing individual differences in emotion regulation tendencies in relation to mindfulness training effectiveness and outcomes.

## Could Pre-Existing Differences in Cognitive Function Affect Mindfulness Training Outcomes?

A rapidly growing body of empirical studies has shown three broad domains of cognitive function are often enhanced following mindfulness training: attention, memory, and executive functions (Chiesa et al., 2011). Among them, attentional control (the ability to control attention), working memory (the ability to maintain and manipulate information over short periods of time for ongoing mental processes), and inhibitory control (the capacity to voluntarily regulate and inhibit prepotent responses) are three representative subdomains that have shown consistent improvement. Although there has been some disagreement regarding whether these constructs are each tapping into dissociable cognitive process or rather a common underlying mechanism, researchers generally agree that individual differences are ubiquitous in nearly all cognitive abilities (Kane and Engle, 2002; Braver et al., 2010; Miyake and Friedman, 2012).

Indeed, individual variability in cognitive function partly appears to be the result of genetic contributions mediated through core neural circuits. Extensive behavioral genetic analyses have found moderate heritability (0.25–0.55) in individual tasks, but this rises to high heritability (0.75) at the level of latent variables, where shared variance across multiple tasks can be extracted to reflect an underlying global process or ability that influences overall performance (Friedman et al., 2008). Similarly, the neural underpinnings of individual differences in attentional control and working memory capacity have been theorized and reliably shown within circuits connected to and centered in the prefrontal cortex (Kane and Engle, 2002; Braver et al., 2010; Burgess et al., 2011). Given these behavioral and biological sources of evidence regarding individual differences in cognitive function, the interesting question with respect to mindfulness training is whether these pre-existing cognitive dimensions of individual variation have important implications for intervention effectiveness and outcomes.

### Attentional Control

Attentional control is one fundamental component of mindfulness training, since cultivating mindfulness requires the ability to control attention in detecting when the mind is wandering, being able to reorient cognitive focus back to the target of concentration, and in sustaining concentration throughout the practice (Lutz et al., 2008; Tang and Posner, 2009). Relatedly, individual differences in self-reported attentional control are also positively correlated with trait mindfulness (Walsh et al., 2009). It is interesting that trait mindfulness, is often referred to as a “naturally occurring” aspect of mindfulness, that is nevertheless often reported to be increased following mindfulness training. These converging lines of evidence indicate that attentional control is indispensable to mindfulness practice, both conceptually and empirically, and further imply that the capacity of attentional control would inevitably exert a dominating influence over the process of mindfulness training. More specially, high attentional control ability at baseline would be expected to bolster mindfulness practices, by minimizing the difficulty of concentrating on present moment experiences, by effectively regulating attentional focus to reduce mind-wandering, and by enabling present moment awareness to be more easily maintained. Consequently, high attentional control ability should thereby result in a more stable mindfulness state that leads to greater training benefits.

Following this line of logic, a number of theoretical accounts of mindfulness training mechanisms have strongly argued for the role of attentional control in subserving training-related cognitive improvements in working memory and inhibitory control (Hölzel et al., 2011; Malinowski, 2013). This theory coincides with prevalent perspectives in cognitive psychology that describe attentional control as a crucial system, through which goals (such as those in cognitive tasks) are actively maintained, monitored, and executed (Kane and Engle, 2002; Posner and Snyder, 1975). In particular, Kane and Engle (2002), as well as Baddeley (2010) have further proposed an attentional mechanism of working memory capacity, based on evidence showing individuals with low span of working memory experience more visual and cognitive interference in tasks (Kane et al., 2001), and are also more susceptible to lures and distractors (Conway et al., 2001). Together, these findings suggest that low span individuals may have substantial amount of difficulty in effectively controlling their attentional focus to support optimal cognitive performance. Additionally, inhibitory control, another major outcome of mindfulness training, has been shown to be partially contingent on attentional control processes that allocate resources for constraining prepotent responses (Howard et al., 2014). Inhibitory control processes are also thought to be heavily involved in supporting working memory, in combination with attentional control, to maintain goal-related information in the face of interference from irrelevant stimuli (Redick et al., 2007). As such, pre-existing individual differences in attentional control are likely to impact the extent of improvement in major cognitive outcomes of mindfulness training, such as working memory and inhibitory control.

While it is logical to assume individuals with high attentional control would gain greater benefits in cognitive function because they can more easily attain a mindfulness state during practices, another important possibility is that of potential ceiling effects in individuals with inherent high levels of cognitive abilities. If an individual is already at the upper limit of his or her attentional control ability, then by theory, this individual would also tend to exhibit an equally high capacity of working memory and inhibitory control, which could indicate that practicing mindfulness would be less likely to result in significant improvement in these three constructs. On the other hand, it is possible that individuals low in attentional control ability may face a more difficult period in the beginning with practicing mindfulness techniques, but would eventually experience more benefits in cognitive function, since they still have room for potential improvement. Indeed, as described earlier, a recent RCT study (*N* = 48) demonstrated that improved working memory capacity following 2 weeks of mindfulness training was mediated by attentional control ability at baseline, such that participants who were initially prone to distraction (low attentional control) exhibited greater benefits in working memory capacity (Mrazek et al., 2013). Therefore, just like emotion regulation, attentional control capacity at baseline could influence the extent of improvement in cognitive function following mindfulness training, but the exact directionality of such influence remains to be ascertained by future studies.

### Working Memory and Fluid Intelligence

Although attentional control and working memory capacity are two overlapping and inter-correlated constructs with shared variance, as demonstrated by studies employing latent variable analyses (Kane and Engle, 2002; Baddeley, 2010), there is also unique variance reflected in each cognitive subdomain (diversity) (Miyake and Friedman, 2012). In fact, inherent differences in working memory capacity may exert their own influence over mindfulness training outcomes through a different mechanism that affects learning abilities. General fluid intelligence is one of the most important factors in learning, which broadly includes problem solving and reasoning abilities that facilitate acquisition of new skills and knowledge (Conway et al., 2003). According to empirical findings, working memory capacity not only correlates highly (0.60–0.80) with level of fluid intelligence (Kane and Engle, 2002), but it also shares common underlying neural substrates with the cognitive processes comprising fluid intelligence (Conway et al., 2003; Burgess et al., 2011). Most importantly, such shared variance between fluid intelligence and working memory may have important implications for mindfulness training, particularly by governing skills acquisition, learning ability, and self-efficacy, which are all pertinent processes for influencing the effects of training (Gully and Chen, 2010). For example, having high working memory capacity may suggest a high level of fluid intelligence. In turn, high fluid intelligence may endow individuals with greater efficiency to learn new skills of mindfulness, and more ability to apply such skills outside of the training context to everyday life, likely resulting in a greater increase the extent of cognitive improvement following mindfulness training. However, it should be noted that the ceiling effects discussed above also have relevance for the present discussion, such that exceptionally high levels of working memory capacity and fluid intelligence at baseline may inevitably result in minimal cognitive changes post-training, due to individuals already hitting the upper boundary of their cognitive abilities.

Overall, the common cognitive outcomes of mindfulness training may reflect inter-correlated relationships, and further, considerable individual variability within each construct. Not only are these baseline individual differences in cognitive function likely to exert reciprocal influences over one another, but they may also interact with the learning and training processes to affect the magnitude of improvement induced by mindfulness training, especially for cognitive outcomes. Therefore, it may be important to take into account pre-existing individual differences in these cognitive constructs when studying mindfulness training effects, in order to more accurately evaluate the extent of improvement among individuals, and conversely, to identify individuals for whom mindfulness training is unlikely to be useful in terms of improving cognitive abilities. It should be noted, however, that there are certainly other cognitive outcome measures of mindfulness training that were not mentioned above, but which could also contribute to differential training effectiveness. The present discussion serves only as a starting point for a more in-depth investigation and conversation regarding the role of individual differences in cognitive function, in terms of their potential impact on mindfulness training effects.

## How Should We Study Individual Differences in Mindfulness Training?

Any attempts to study these promising effects of individual differences on mindfulness training outcomes must carefully consider common methodological issues and constraints that have been emphasized in individual differences-based studies in psychology. To facilitate future investigation, the following section describes several general theoretical and empirical considerations and addresses potential methodological challenges with suggestive solutions. We also provide an empirical example as an illustration of how an individual differences perspective could influence future mindfulness research investigations. Finally, we discuss potential research strategies for assessing the efficacy of mindfulness-based interventions that are tailored to individuals’ characteristics.

### Sample Size

The first issue to consider in research on individual differences is the need for a large enough sample size to have sufficient statistical power to detect individual difference effects. Unfortunately, the sample size in the majority of mindfulness training studies typically fall within the range of 10–50 people per group, with the exception of a few recent large-scale studies, that reach more than 50 participants per group (Kuyken et al., 2013; Engert et al., 2017; Hildebrandt et al., 2017). In fact, the issue of small sample size has long been criticized within mindfulness research, though not for the reason of individual differences; instead, the concerns are mostly oriented toward the over-inflation of reported training effects and the generalizability of such results (Baer, 2003; Van Dam et al., 2018). Regardless of whether researchers are interested in making inferences about population-level or individual-level training effects, a large sample size is necessary to capture a wide range of inter-subject variability from the population, and to have sufficient statistical power to detect effects that are likely to be more subtle in nature (Goldberg et al., 2017; Van Dam et al., 2018).

However, it is worth acknowledging that acquiring data with large sample size is not easily accomplished in psychological intervention research, due to several competing empirical constraints. First, having more than 25 participants per training group could potentially jeopardize the training quality, especially if there is only one instructor assigned to provide instructions, respond to questions, and monitor the progress of each participant. For this reason, previous studies have mostly adopted a sample size of under 30 per group for mindfulness training studies, which means that to accumulate a sufficiently large sample size requires a greater outlay of time and resources. Yet, there are a few alternative solutions that can increase the overall sample size without, overly increasing the time required for data collection: (1) running multiple training groups in parallel with multiple instructors (one per group); and (2) having more teaching assistants with one instructor to guide a larger training group. The former option is an ideal solution when there are adequate financial and instructional resources to support such large-scale study. The latter option would likely reduce the financial burden more than the former, but it is currently an unstudied question as to whether or not having trained teaching assistants would introduce unknown effects to training quality.

A related issue is that of participant attrition. This factor, which is common in intervention research, also makes collecting large sample size data challenging, especially for the types of longitudinal designs becoming more common in mindfulness training research, which require a significant time commitment and persistence from participants throughout the study period. This challenge may be potentially addressed by offering greater incentives for study completion or by providing easily accessible training programs to participants via online platforms. Indeed, web-based mindfulness programs have increasingly been developed and distributed in recent years, but the standardization and effectiveness of such programs warrants further investigation. In particular, a recent review suggests some support for the efficacy of web-based mindfulness training programs on improving psychological well-being, with most studies showing large effect sizes (Fish et al., 2016). Yet methodological concerns including selection bias and lack of control group make it difficult to evaluate and compare the efficacy of these online programs with other web-based health enhancement alternatives.

### Reliability and Validity

The second issue to consider is the reliability and validity of measurement tools used for examining mindfulness training effects. Reliability refers to the overall consistency of a measure to produce similar results when administered multiple times in the same individual when all other factors are held constant, whereas validity is the extent to which an assessment measures what it is supposed to measure. While both concepts are fundamental to psychometric theory and are valued highly in psychology and cognitive neuroscience research, they are especially worth underscoring for studies adopting an individual differences approach. Validity is generally less of a concern if the construct of interest is clearly defined and operationalized by the measurement tools, based on established theories, and more importantly, if the measurement tools have previously been examined in empirical studies for validity. However, Van Dam et al. (2018) have pointed out the semantic ambiguity in defining the construct of mindfulness, specifically within the realm of mindfulness research. They emphasized how this can lead to problems of construct validity that are particularly acute when considering the different self-report questionnaires of mindfulness, that incorporate various semantic associations that are commonly being used as outcome measures of mindfulness training (Black et al., 2012; Goldberg et al., 2016). Moreover, this issue becomes especially problematic when multiple studies are compared across various dimensions to draw general conclusions regarding effects of training on a construct with a wide range of different semantic definitions. Therefore, investigators should explicitly address the issue of validity in mindfulness research, by being extremely clear about the operationalized definitions of all constructs of interests measured in the study, especially that of mindfulness.

Reliability plays an even more pivotal role in individual differences research, by directly influencing the extent to which individual differences effects can be observed in studies, as well as the stability of such effects, if any. Intuitively, low measurement reliability could lead to fluctuating results, since the assessment would yield fairly different scores each time it is administered to the same individual. For example, low reliability can lead to unstable correlational relationships between the measured variables, resulting in inconsistent conclusions about individual differences in these variables. Another notable, perhaps more serious consequence arises from the reliability paradox, which has recently been shown to be especially pernicious with regard to robust cognitive paradigms. Here, well-established experimental effects are consistently replicated at the group level, simply because these tasks are less sensitive to between-subject variability (Hedge et al., 2017).

The reliability paradox is problematic because most studies of individual differences employ a correlational approach for data analysis; yet if seemingly well-validated tasks that nevertheless have low between-subject variability are employed, it can greatly undermine the correlational relationships observed between theoretically important variables, resulting in misleading conclusions. As a concrete example, the cognitive tasks that are frequently utilized to assess mindfulness training effects on attentional control and related constructs (e.g., Stroop, Flankers, SART), are precisely the ones demonstrated by Hedge et al. (2017) to have rather low test-retest reliabilities, typically less than the conventional standard of 0.70 adopted in psychometric research. Thus, if and when the focus of mindfulness research shifts toward an individual difference perspective, it will be imperative for researchers to be highly aware of such problems in the cognitive paradigms that may be employed, and to avoid potential pitfalls by exercising caution in study planning and task selection.

Although the abovementioned issues are troublesome for individual differences research in mindfulness training, two straightforward solutions could potentially remedy some of the negative impacts. The first solution is to compute and report reliability coefficients for each assessment (behavioral and neuroimaging), and for each new sample, by following guidelines from the psychometric literature (Braver et al., 2010; Cooper et al., 2017; Hedge et al., 2017; Parsons et al., 2018). This quality control step would qualitatively evaluate the extent to which the observed effects can be trusted, adding another source of validation and objective reference when reporting study results. The second straightforward solution is to carefully select assessment tools with excellent reliability (>0.8 reliability coefficient) based on prior research, or measures with at least moderate reliability (0.6–0.7), when there are no other alternatives for measuring the specific construct of interest (Hedge et al., 2017).

Furthermore, it is worth mentioning that for individual differences research, it is imperative to select measurement tools that not only have good reliability but also are sensitive to inter-individual variability. As described above, it is not logically entailed that a paradigm yielding consistent experimental effects at the group-level will also capture unique variance between individuals. Fortunately, there has been new progress in the development of cognitive task batteries sensitive to both group and individual level effects (Braver, 2012; Cooper et al., 2017), and some individual-focused batteries are being adapted for on-line testing (Hicks et al., 2016). Together, these should gradually provide more new avenues for assessing cognitive effects in individual differences-based studies of mindfulness training.

### An Example: Individual Preferences in Mindfulness Training

Identifying predictors or moderators of mindfulness training effects is one approach to investigate the question of individual differences. However, one can also tackle individual variability in response to mindfulness training from a slightly different angle. Previous reports have shown that individuals, especially patients who undergo psychological intervention, have preferences for at least one aspect of their intervention and if such preferences were not met during the intervention, poorer outcomes are observed at post-intervention (Williams et al., 2016). Additionally, one study (*N* = 247) also established the presence of individual preferences for specific meditation techniques, but not all techniques, taught within a training program, suggesting that people may not find every training technique to be helpful and may only engage in techniques they find most effective (Burke, 2012). Similarly, preferences for specific modality of mindfulness practice anchors (e.g., using breath, imagery, or auditory-phrase as a focus of attention), were found among novices (*N* = 117) who practice mindfulness and such preferences also underwent change over the course of mindfulness training in some individuals (Anderson and Farb, 2018). Unfortunately, most mindfulness training programs tend to present students with a multi-faceted package, encompassing a broad set of different mindfulness techniques, while overlooking the fact that individual preferences may exist with regard to the techniques that are taught; such preferences may have the potential to influence the outcomes of mindfulness training.

Building upon this work of preferences in psychological intervention, we recently conducted a study examining individual preferences in mindfulness training, specifically focusing on whether or not individual differences in personality traits can predict personal preferences for practicing one specific mindfulness technique over the other alternatives (Tang and Braver, 2020, PsyArXiv). Relevant dispositional traits, including but not limited to personality, trait mindfulness, emotion profiles, and empathy were assessed among a group of meditation-naïve participants who were exposed to four different mindfulness sessions, via a novel on-line protocol, with each session involving exposure to one of the following four commonly practiced techniques – focused attention, open monitoring, loving kindness, and body scan. Among all four techniques, preferences for open monitoring and loving kindness were found to be predicted by dispositional traits, such that the tendency to rank open monitoring as the most preferred technique was associated with higher level of non-judgment, a facet of trait mindfulness. Conversely, a greater preference for loving kindness was related to higher level of empathic concerns and perspective taking, two facets of empathy. Likewise, participants self-reported stronger mindfulness states as assessed via the State Mindfulness Scale when practicing with their preferred technique, suggesting that individual preferences for specific techniques may lead to differential training outcomes.

This finding from our own work, together with previous reports of individual preferences for different meditation techniques (Burke, 2012) and mindfulness practice anchors modality (Anderson and Farb, 2018) still leave open the question of how such preferences might impact the outcomes of mindfulness training. If individual preferences do indeed have substantial contribution to differential training outcomes, then such information would be critical for effectively applying and tailoring existing mindfulness programs toward individual characteristics, as a means to enhance their overall effectiveness and cost-effectiveness in achieving desirable outcomes. Furthermore, this also leads to another important research direction concerning the outcome and efficacy assessment of mindfulness programs that are tailored to individual characteristics.

One logical first step in approaching this question would be a prospectively designed empirical study that, in the pre-test phase, gathers information about individual characteristics and traits to make predictions about individuals’ preferences for specific mindfulness practices, as well as asking questions that directly probe their general preferences for relevant intervention components, such as delivery format and settings. Once this information is compiled for each individual, researchers are able to assign individuals either to mindfulness practices and programs that are compatible with their preferences and needs, or ones that are incompatible. Consequently, such a prospective design would allow a direct comparison of potential improvement in the targeted outcomes between the two groups, enabling strong inferences about interventions efficacy as a function of intervention compatibility based on individual characteristics. Finally, as more attention and research effort are devoted to individual differences investigation in mindfulness training, more innovative approaches for assessing the effectiveness of individual-tailored mindfulness-based interventions would become available. Our discussion only seeks to provide a concrete example of how to examine individual differences in mindfulness training, and how to build upon these research findings to rigorously evaluate the outcomes of individual-oriented interventions programs.

## Future Directions

In this review, we have primarily focused on behavioral studies of individual differences related to mindfulness training, but it is worth noting that studies of mindfulness training effects utilizing cognitive neuroscience approaches, though still in their infancy, would also benefit from adopting an individual differences perspective in future investigation. An impressive body of evidence has demonstrated inter-individual variability in the brain using neuroimaging methods (e.g., fMRI), acquired during both task-evoked and resting states (Finn et al., 2015; Dubois and Adolphs, 2016; Gordon et al., 2017a; Gratton et al., 2018), with the latter echoing findings from the older EEG literature demonstrating individual-differences in frontal asymmetries (e.g., Wheeler et al., 1993; Davidson et al., 2003).

Brain functional connectivity profiles provide a particularly clear example. In particular, connectivity profiles in task and resting states can act as a “fingerprint” that reliably distinguishes individuals from rest of the group (Finn et al., 2015). Likewise, Gratton et al. (2018) illustrated that task-evoked modulation of functional networks primarily behaves in an individual-specific manner in the frontoparietal regions relevant for high-level cognitive processes. These regions, along with the default mode network manifested in resting state, are found to be affected by mindfulness training (Tang et al., 2015), indicating that an individual differences perspective would be highly meaningful for exploring the effects of mindfulness training on the brain.

Neuroimaging studies of individual differences are strongly benefited by a longitudinal design with multiple sessions of scanning data per participant, to most accurately identify features unique to the individuals (Gordon et al., 2017b). Fortunately, similar longitudinal research designs (though with fewer sessions) are required in mindfulness training research to compare pre- and post-training changes in brain and behavior. Therefore, exploiting these existing strengths for the purpose of investigating individual differences is not only convenient but also may greatly enrich the scientific understanding of mindfulness training in an unprecedent way.

Relatedly, recent advances in neuroimaging analytic approaches also hold promise for interrogating individual differences within the context of mindfulness training. Some of these approaches include but not limited to: (1) structural equation modeling, which can directly model between-subject variability and is sensitive to the complexities of brain-behavior relationships, as well as the psychometric properties of data critical for individual differences-type analysis (for a detailed review, see Cooper et al., 2019); (2) multi-variate pattern analysis (MVPA) approaches, which can be used to decode different mental states during mindfulness training based on distributed patterns of brain activity, and which may be especially powerful in detecting and identifying differences in mental states across individuals who practice mindfulness (Weng et al., 2018); and (3) network analysis (i.e., graph theory), which captures the functional organization of large-scale brain networks through measures of network properties that can explain individual differences in cognition and behavior (for a detailed review, see Tompson et al., 2018), and which is also beginning to be used to study brain-related changes following mindfulness training (Gard et al., 2014). Bridging these new methodologies in future investigation may further deepen our current knowledge regarding the brain mechanisms underlying mindfulness training and associated inter-individual variability.

Finally, the personalized medicine and patient-centered care movements suggest a completely new direction of research that affords tremendous opportunities and progress for bridging psychology and health science. In particular, mindfulness research has yet to investigate the feasibility of modifying and tailoring existing established prevention and treatment programs to individual’s characteristics and needs. Although standardized programs enable a widespread implementation of intervention protocols in an easily controlled and accessible manner, customizing these multifaceted packages could potentially be illuminating at the individual level. For instance, prior work (Burke, 2012; Anderson and Farb, 2018; Tang and Braver, 2020, PsyArXiv) have already shown that individuals may not find every component of a mindfulness training program to be helpful, and that such preferences can be predicted by personality traits. Furthermore, there is also evidence from a recent survey study (*N* = 500) indicating individual preferences for certain delivery formats (group, internet, one-on-one) of mindfulness training (Wahbeh et al., 2014), which could potentially influence how well one practices and learns the training. Therefore, these findings, in combination with existing evidence of individual differences in intervention effectiveness, call for more research and development of individual-specific intervention programs.

## Conclusion

As this review has highlighted, evidence is accumulating that individual differences in dispositional traits, psychological well-being, and cognitive function could play a critical role in contributing to the heterogeneity in mindfulness training effects across individuals. Consequently, moving toward an individual difference perspective is imperative for accurately evaluating mindfulness training effects and outcomes to inform subsequent application in real-world settings. In this review, we discussed putative traits and characteristics relevant to mindfulness training effectiveness, as well as theoretical considerations and useful examples for future investigations on this topic. It is our hope that researchers will not only be made aware of these critical issues associated with individual differences research within the context of psychological intervention, but will also be encouraged to explore various ideas and approaches to tackle this emerging question in mindfulness training research.

## Author Contributions

RT contributed to the conception, writing, and critical revision of the manuscript, and final approval of the version to be published. TB contributed to the writing and critical revision of the manuscript, and final approval of the version to be published.

## Conflict of Interest

The authors declare that the research was conducted in the absence of any commercial or financial relationships that could be construed as a potential conflict of interest.
